# Preparation of Zeolite A from Ion-Adsorbing Rare Earth Tailings for Selective Adsorption of Pb^2+^: An Innovative Approach to Waste Valorization

**DOI:** 10.3390/molecules29215065

**Published:** 2024-10-26

**Authors:** Qiangwei Huang, Wenbo Wang, Wenhui Lai, Binjun Liang, Bin Xiao, Jihan Gu, Zheyu Huang, Xiangrong Zeng, Hui Liu, Haixiang Hu, Weiquan Yuan

**Affiliations:** 1 School of Resources and Civil Engineering, Gannan University of Science and Technology, Ganzhou 341000, Chinaliangbinjun1205@163.com (B.L.); gujihan@gnust.edu.cn (J.G.); hzy_gnust@163.com (Z.H.); zengxr986@163.com (X.Z.); 9320220005@gnust.edu.cn (H.L.); 2 Ganzhou Key Laboratory of Mine Geological Disaster Prevention and Control and Ecological Restoration, Ganzhou 341000, China

**Keywords:** ion-adsorbing rare earth tailings, classification activation, zeolite A, adsorption performance

## Abstract

Ion-adsorbing rare earth tailings (IRETs) contain a large amount of clay minerals, which are a potential source of silicon and aluminum for the preparation of zeolite materials. The complexity of the tailings’ composition and the impurity composition are the main difficulties in the controllable preparation of zeolite. Herein, IRETs were treated by classification activation technology for the preparation of IRET-ZEO, which was used for the removal of heavy metal Pb^2+^ in water. A new method of resource utilization of ion-type rare earth tailings is realized by “treating waste with waste”. The results showed that the IRETs were classified and then thermally activated, and the optimal activation parameter was calcination at 850 °C for 1 h. The optimal NaOH concentration used in the crystallization process was 5 mol/L, with a crystallization time of 3 h and a crystallization temperature of 85 °C, and the crystallization product was zeolite A. The removal rate of the Pb^2+^ solution with an initial concentration of 100 mg/L was as high as 96.7% in an acidic solution with a pH value from 2 to 5.5. In particular, when the solution pH was higher than 4.2, the adsorption rate of Pb^2+^ was close to 100%. The IRET-ZEO showed a fast adsorption rate (5 min to reach adsorption equilibrium), a large adsorption capacity (378.35 mg/g), excellent acid resistance, and selectivity and regenerability for Pb^2+^. This work provides a new strategy for the green resource utilization of IRETs and the treatment of lead-containing wastewater.

## 1. Introduction

Heavy metal ion contamination is currently one of the most significant forms of water pollution, posing a notable threat to both ecological safety and human health [1]. Large volumes of wastewater produced during mineral extraction and processing contain heavy metal ions such as Cu^2+^, Pb^2+^, Cd^2+^, and Cr^2+^. Once these heavy metal ions enter the aquatic environment, toxic heavy metal ions enter the food chain and accumulate in the human body, which are difficult to eliminate naturally [2]. Currently, the primary methods for treating heavy metal ions include chemical precipitation, biological methods, ion exchange, membrane separation, and adsorption [3,4,5]. However, chemical precipitation requires substantial amounts of chemicals and may lead to secondary pollution [6]; biological treatment is difficult to control [7]; the ion-exchange method has lower regeneration efficiency of the ion-exchange agents [8]; and membrane separation is costly and associated technologies are still being developed [9]. As environmental protection standards become increasingly stringent, the development of novel, green, and efficient technologies for treating heavy-metal-ion-contaminated wastewater is imperative. Among these methods, adsorption is distinguished by low cost, simple operation, the absence of secondary pollution, and high efficiency [10,11,12,13]. Consequently, the development of high-performance adsorbent materials has emerged as a major research focus.

Zeolite materials are known for their large specific surface area, unique pore structure, and ease of modification, which makes them widely used in adsorption, separation, and catalysis [14]. Compared to natural zeolites, synthetic zeolite provides advantages such as higher purity, uniform particle size, controllable morphology, and superior performance. At present, the methods for synthetic zeolite can be divided into three categories based on the type of raw materials used: the first method uses pure chemical reagents as the source of silica and alumina for zeolite material synthesis; the second method employs waste containing silicon and aluminum as the raw material, which undergoes pre-activation treatment prior to use in zeolite synthesis; and the third method combines waste with a small amount of chemical reagents for material synthesis. The zeolite prepared by the first method has consistent physicochemical properties and the greatest level of control is achieved during this synthesis process. However, it requires a large consumption of chemical reagents, resulting in high production costs and the potential generation of toxic by-products during synthesis. In contrast, the latter two methods effectively utilize the silicon and aluminum components in the waste, significantly reducing production costs and fulfilling the objective of turning waste into valuable resources [15].

The use of solid waste as a raw material for zeolite synthesis is an important approach to the resource reutilization and high-value utilization of waste [16,17,18,19]. Common types of solid waste used are electrolytic manganese residue [20], crushed stone [21], waste glass [22], fly ash [23], solar panels [24], and lithium leach residue [25]. Existing research mainly focuses on utilizing the silicon and aluminum components in solid waste. This process involves activating and leaching the inert silicon and aluminum components in the waste before using them for zeolite material synthesis [26]. Generally, the synthesis of zeolite from tailings requires a complex multi-step process, including “activation-silicon and aluminum dissolution-polymerization and zeolitization” [27]. However, this process is characterized by issues such as reaction complexity, stringent reaction conditions (high temperature and pressure), high consumption of acids and bases, and significant energy consumption [28].

Ion-adsorbing rare earth ores (also known as weathered crust elution-deposited rare earth ores) are primarily distributed across southern China. Due to their unique mineral occurrence characteristics, in situ leaching is widely adopted for extraction [29]. However, this method entails considerable risks of geological disasters, such as landslides, and poses substantial challenges to ecological safety. The tailings from ion-adsorbing rare earth mines mainly consist of minerals such as quartz, clay, mica, and feldspar [30,31], which are abundant in silicon and aluminum components. These tailings serve as potential sources of silicon and aluminum for the preparation of zeolite and other mineral materials. Yang et al. [32] successfully synthesized 33 types of functionalized red pigments from IRETs subjected to mechanical grinding and high-temperature calcination by doping with iron and lanthanum elements. Zhou et al. [30] treated ion-adsorbing rare earth mine tailings to study the adsorption of La^3+^ and Y^3+^. Kinetic studies showed that the adsorption process of La^3+^ and Y^3+^ onto the treated tailings follows a pseudo-second-order kinetic model. In the field of zeolite synthesis from tailings, Cheng et al. [33] activated the silicon and aluminum components in the tailings via an alkali fusion method, followed by hydrothermal synthesis with additional aluminum species to produce zeolite A. This material exhibited excellent adsorption performance towards typical pollutants from rare earth mining operations, such as Pb^2+^, Cd^2+^, Cu^2+^, NH_4_^+^, PO_4_^3−^, and F^−^. Current studies involve stringent reaction conditions for the synthesis of zeolite A, leading to high energy consumption and poor control over product quality. These problems primarily stem from the significant variability in the reactivity of different silicon and aluminum minerals, posing challenges for the efficient utilization of the tailings through a single activation and crystallization process. Based on the difference in particle size distribution of the components in the IRETs, different active minerals are fractionally activated by grading, which can be used for the synthesis of different types of zeolite materials, such as A-type zeolite and high-silica zeolite. The use of IRETs for zeolite preparation based on classification activation technology has not been reported in the previous literature.

In this work, silicon–aluminum minerals from IRETs were employed as raw materials, and a classification activation method was utilized to separate the tailings into highly active clay-rich components and inert silica-rich (quartz) components. This work focused on the highly active clay-rich components, using thermal activation to stimulate the reactivity of the clay minerals and facilitate the utilization of the high-activity silicon and aluminum components in the zeolitization process. Based on experimental conditions, a straightforward, cost-effective method was developed for the preparation of IRET-based zeolite (IRET-ZEO), which exhibited excellent adsorption performance. The adsorption performance of IRET-ZEO for Pb^2+^ was thoroughly investigated. The effects of solution pH, IRET-ZEO dosage, adsorption time, and initial concentration of Pb^2+^ on adsorption behavior were examined, and the optimal adsorption conditions were determined. Additionally, adsorption kinetics, competitive adsorption, and cyclic regeneration performance were also analyzed. Therefore, this study offers a new strategy and significant practical potential for the green resource utilization of IRETs and their application in the treatment of heavy-metal-containing wastewater.

## 2. Results and Discussion

### 2.1. Particle Size Distribution and Composition of IRETs

**Figure 1:**
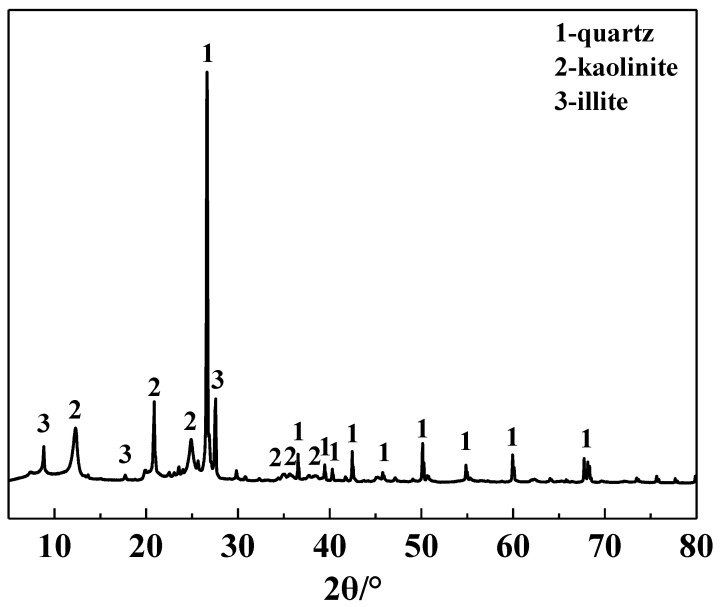
XRD pattern of IRETs.

**Figure 2:**
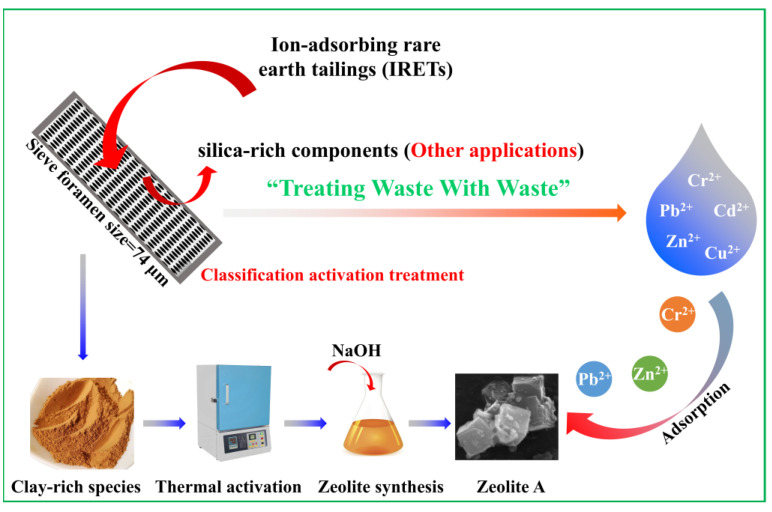
Schematic diagram of resource utilization for IRETs.

**Figure 3:**
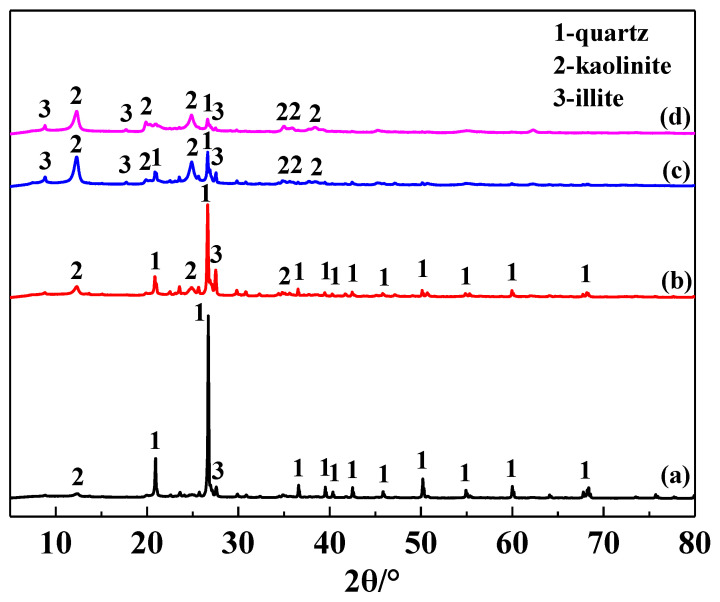
XRD pattern of different particle sizes of IRETs ((a) >0.8 mm, (b) 0.074~0.8 mm, (c) 0.044~0.074 mm, and (d) <0.044 mm).

The main chemical composition of the IRETs is provided in Table 1. The content of SiO_2_ was highest in the IRETs, followed by Al_2_O_3_, with the combined content of the silicon oxides and aluminum oxides accounting for 86.46%. The concentrations of other oxides were relatively low. The XRD pattern of the IRETs is shown in Figure 1, indicating that the dominant mineral present in IRETs is quartz, along with small amounts of kaolinite and illite. Therefore, these rare earth tailings serve as an adequate source of Si and Al for the synthesis of zeolite materials. The schematic diagram of resource utilization for IRETs was shown in Figure 2. The particle size distribution of the IRETs is shown in Table 2. The particle size distribution of the tailings is uneven, with a predominance of coarse particles. Particles larger than 0.8 mm accounted for 57.00% of the total, while the smallest particle size components, 0.044 mm to 0.074 mm, accounted for only 2.44%. The XRD analysis of each particle size component of the IRETs (Figure 3) revealed that the main mineral phases are quartz, kaolinite, and illite [32]. The clay minerals, kaolinite and illite, are primarily concentrated in particles smaller than 0.074 mm, whereas quartz is mainly distributed in the coarser particles. Therefore, screening and classification can effectively separate the silica-rich quartz components from the clay minerals, facilitating the subsequent classification and activation process.

### 2.2. Effect of Crystallization Conditions on the Phase of IRET-ZEO

**Figure 4:**
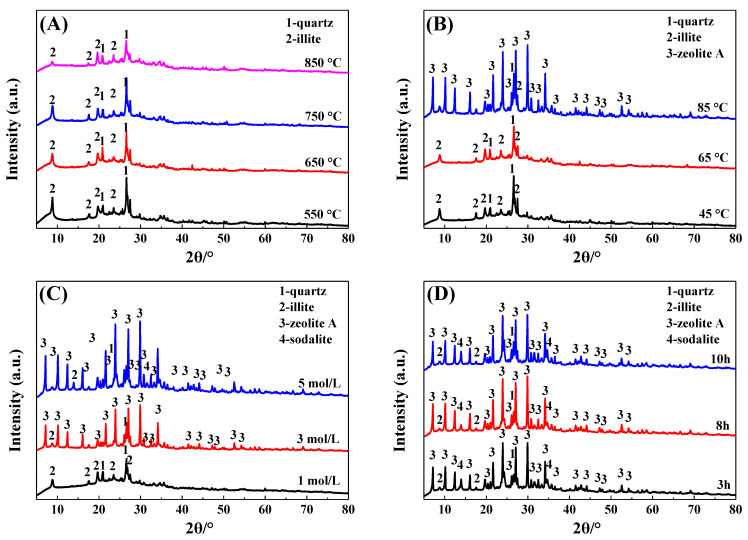
Effect of different crystallization conditions on the physical phase of IRET-ZEO ((**A**) thermal activation temperature; (**B**) crystallization temperature; (**C**) alkali concentration; and (**D**) crystallization time).

The effects of thermal activation temperature, crystallization temperature, alkali concentration, and crystallization time on the phase of the IRET-ZEO are shown in Figure 4. As can be seen from Figure 4A, the thermal activation temperature of the IRETs had little effect on the physical phase composition of the zeolite products, the characteristic diffraction peaks of the kaolinite in the activated products at each temperature disappeared and transformed into an amorphous structure, and its reactivity was significantly improved. The characteristic diffraction peak of illite gradually decreased with the increase in calcination temperature. In order to obtain better crystallization activity of the IRETs, 850 °C was chosen as the optimal calcination temperature.

When the crystallization temperature was lower than 65 °C, no obvious zeolite phase appeared in the crystallization product. This might be due to the sufficient depolymerization of the silica and aluminum components. When the crystallization temperature reached 85 °C, obvious A-type zeolite characteristic diffraction peaks appeared in the product, which indicated that the silica–aluminum component in the IRETs crystallized to form the A-type zeolite phase at this crystallization temperature. Therefore, 85 °C was chosen as the optimal crystallization temperature (Figure 4B).

In order to study the influence of the alkali concentration of the crystallization reaction on the phase composition of zeolite in the crystallization products, the phase compositions of the crystallization products of activated tailings in 1 mol/L, 3 mol/L and 5 mol/L NaOH were investigated. As can be seen from Figure 4C, the characteristic diffraction peaks of zeolite in the crystallization products were gradually enhanced with the increase in alkali concentration, indicating that the crystallinity of zeolite in the crystallization products increased significantly with the increase in alkali concentration, and therefore 5 mol/L was selected as the optimal alkali concentration.

**Figure 5:**
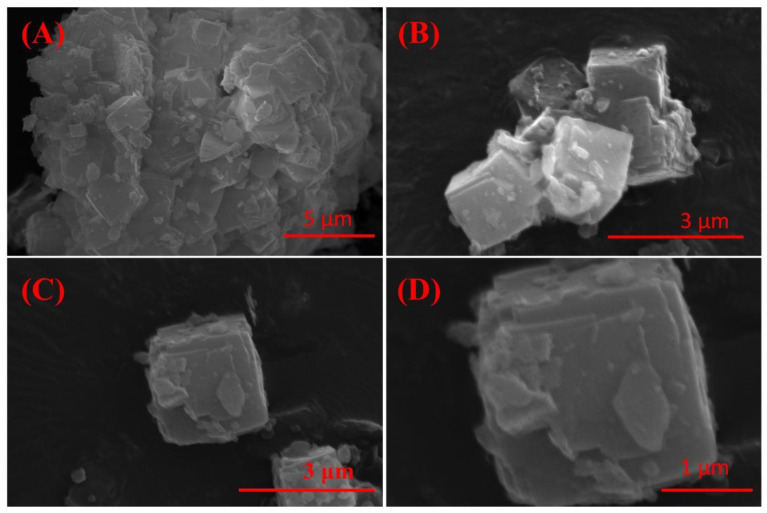
SEM image of IRET-ZEO with different magnifications (**A**–**D**) (activation temperature was 850 °C, activation time was 1 h, NaOH concentration was 5 mol/L, crystallization time was 3 h, and crystallization temperature was 85 °C).

The effect of crystallization time on the physical phase of the crystallization products is shown in Figure 4D; with the increase in crystallization time, the phase of the crystallization products was changed insignificantly. Moreover, considering the time cost of synthesis, 3 h was chosen as the optimal crystallization time. The SEM image (Figure 5) of the crystallization product showed that its micro-morphology was dominated by cubic A-type zeolite, which was consistent with the XRD results. And there were obvious defects on the surface of the zeolite, which provided abundant action sites for the adsorption of heavy metal ions, which was conducive to the improvement of the adsorption performance of the material.

### 2.3. Effect of Factors on Adsorptive Properties of Synthesized Zeolite

**Figure 6:**
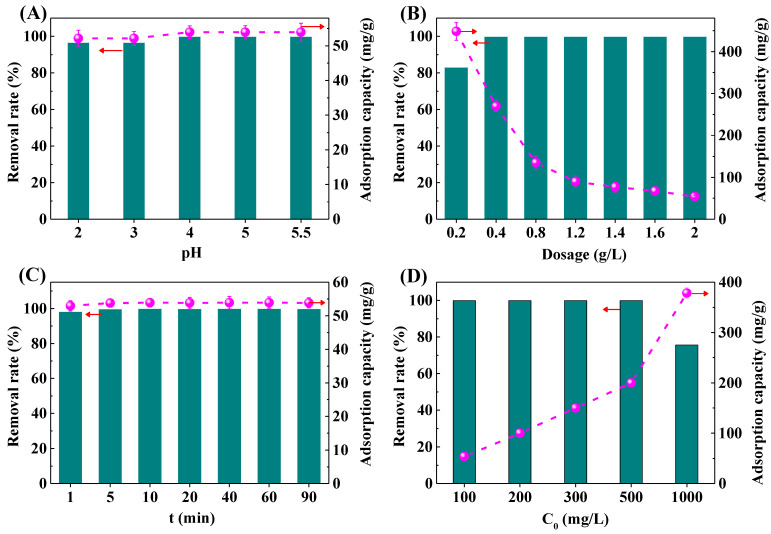
Removal rate and adsorption capacity of Pb^2+^ by IRET-ZEO as a function of pH (**A**), adsorbent dosage (**B**), contact time (**C**), and initial concentration (**D**) in the solution.

The solution pH is one of the important factors for the adsorption performance of the materials [34]. The removal rate and adsorption capacity of Pb^2+^ by the IRET-ZEO under different pH conditions is shown in Figure 6A. In the acidic solution with pH 2–5.5, the removal rate of the IRET-ZEO for Pb^2+^ with an initial concentration of 100 mg/L was higher than 96.72%. Especially when the pH was higher than 4.0, the concentration of Pb^2+^ was reduced by the IRET-ZEO from the initial 100 mg/L to less than 0.0020 mg/L, and the adsorption capacity of Pb^2+^ was 53.91 mg/g. The removal rate and adsorption capacity of Pb^2+^ under different pH conditions are shown in Figure 6A. This indicates that the IRET-ZEO showed excellent acid resistance under a wide range of pH conditions, and the IRET-ZEO exhibited a greater potential for application in the removal of Pb^2+^ from drinking water and Pb^2+^-containing wastewater with a low concentration of Pb^2+^.

Figure 6B shows the removal rate and adsorption capacity of Pb^2+^ under different dosages of the IRET-ZEO. When the dosage of the IRET-ZEO was 0.2 g/L, the removal rate of the Pb^2+^ solution with an initial concentration of 100 mg/L was 83.11%, and with the increase in the IRET-ZEO dosage, the removal rate of Pb^2+^ also increased. When the dosage of the IRET-ZEO was more than 0.4 g/L, the removal rate of Pb^2+^ was close to 100%. This was due to the increase in the capacity of the IRET-ZEO, which increases the adsorption sites and the surface area of the IRET-ZEO to achieve a better adsorption effect. The adsorption of Pb^2+^ by the IRET-ZEO decreased with the increase in dosage, and an adsorption capacity of 448.05 mg/g was achieved at a dosage of 0.2 g/L, which confirmed that the IRET-ZEO exhibited an excellent adsorption capacity of Pb^2+^.

The effect of different adsorption times on the removal rate and adsorption capacity of Pb^2+^ is shown in Figure 6C. After 1 min of adsorption, the removal rate of Pb^2+^ by the IRET-ZEO was 98.2% in the solution with an initial concentration of 100 mg/L, and the adsorption capacity was 52.94 mg/g. With the increase in adsorption time, the adsorption capacity of Pb^2+^ by the IRET-ZEO reached equilibrium quickly, in 5 min, which indicated that the IRET-ZEO displayed a faster adsorption rate for Pb^2+^.

In order to explore the adsorption capacity of the IRET-ZEO, the effects of different initial concentrations of Pb^2+^ on the removal rate and adsorption capacity are shown in Figure 6D. When the initial concentration of Pb^2+^ was less than 500 mg/L, the removal rate of the IRET-ZEO of Pb^2+^ was higher than 99% in all cases, and the removal rate remained at 75.67% when the initial concentration of Pb^2+^ was 1000 mg/L. The adsorption capacity of the IRET-ZEO of Pb^2+^ at this time reached 378.35 mg/g, which might be owing to the abundant surface sites and pore structure properties of the IRET-ZEO. A comparison of the adsorption performance of IRET-ZEO for Pb^2+^ with other adsorbents in previous literature is summarized in Table 3. IRET-ZEO exhibited significant advantages in adsorption capacity, adsorption rate, and synthetic cost compared with other adsorbents in the previous literature. This indicated that IRET-ZEO exhibited excellent adsorption capacity when treating solutions containing high initial concentration of Pb^2+^, and showed an excellent potential in practical Pb^2+^-containing wastewater treatment.

### 2.4. Adsorption Kinetics

**Figure 7:**
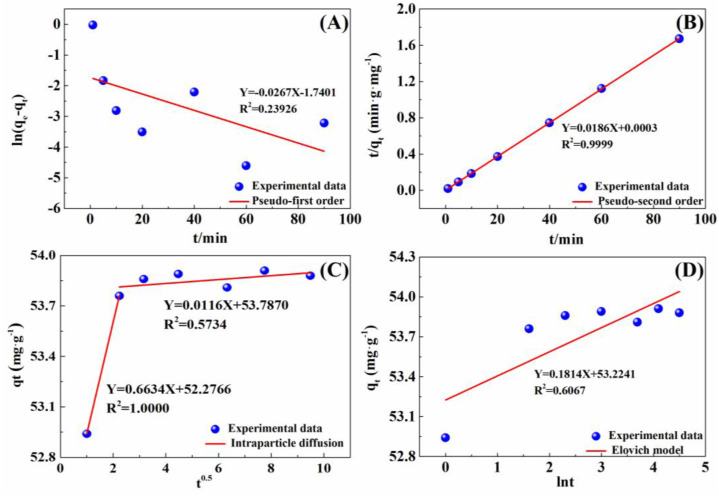
Adsorption kinetics of Pb^2+^ on IRET-ZEO plotted using (**A**) pseudo-first order, (**B**) pseudo-second order, (**C**) intraparticle diffusion, and (**D**) Elovich equation. Adsorption conditions: C_0_ was 100 mg/L; pH was 4; dosage was 0.2 g/L; and volume of solution was 50 mL.

The adsorption kinetics of the IRET-ZEO to Pb^2+^ were investigated using the pseudo-first-order kinetic model, pseudo-second-order kinetic model, Elovich kinetic model, and intraparticle diffusion model. The results are shown in Figure 7, and the corresponding kinetic parameters are listed in Table 4. The linear fit of the pseudo-second-order kinetic model (R^2^ = 0.9999) was markedly superior to that of the other models, indicating that the adsorption of Pb^2+^ by the IRET-ZEO follows the pseudo-second-order kinetic model, suggesting that the adsorption process is controlled by chemical interactions. The rate constant k_2_ obtained from the pseudo-second-order model was 1.3486 g·mg^−1^·min^−1^, demonstrating a remarkably rapid adsorption rate. The equilibrium adsorption capacity q_e_ was 53.76 mg/g, closely matching the experimental value of 53.88 mg/g, further confirming the accuracy of the adsorption kinetic model. In addition, the linear fit of the intraparticle diffusion model showed that the adsorption process was not influenced by a single diffusion factor but consisted of a rapid adsorption process and an adsorption process that tended toward saturation. The efficiency and cost in the practical application of the adsorbent was determined by the faster adsorption kinetics [43], which was one of the important parameters for the practical application of adsorption materials.

### 2.5. Selective and Regeneration Adsorption

**Figure 8:**
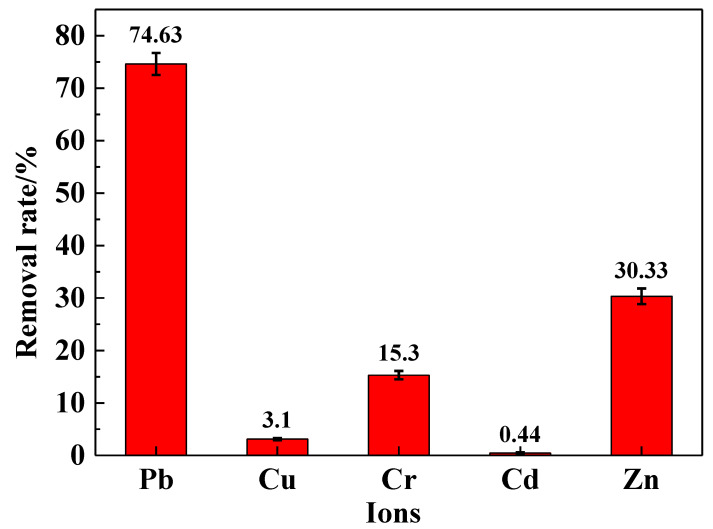
Removal rate of metal ions in mixed solution by IRET-ZEO.

To investigate the selective adsorption rate of the IRET-ZEO in complex metal ion solutions, its adsorptive behavior was studied in a mixed solution containing five coexisting metal ions. The results are shown in Figure 8. In the mixed ion solution, the IRET-ZEO achieved a removal rate of 74.63% for Pb^2+^. This was followed by the removal of Zn^2+^ and Cr^3+^, with removal rates of 30.33% and 15.3%, respectively. The adsorption performance of the IRET-ZEO for Cu^2+^ and Cd^2+^ in the mixed solution was less effective. The IRET-ZEO maintained strong adsorption performance for Pb^2+^ regarding the complexity of the metal ion mixture, demonstrating excellent selective adsorption capabilities and excellent adsorption potential for complex solutions.

**Figure 9:**
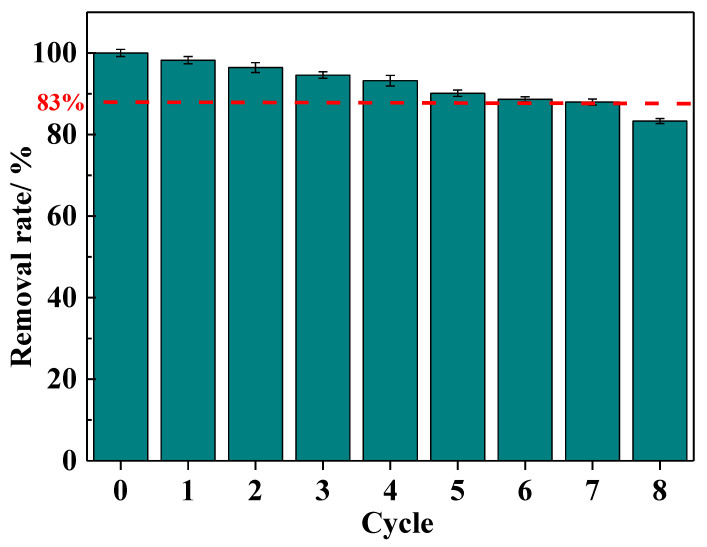
Regeneration performance of IRET-ZEO.

The regeneration adsorption performance of the IRET-ZEO was also studied (Figure 9). The results showed that with increasing regeneration cycles, the Pb^2+^ removal rate gradually decreased, which could be attributed to the partial destruction of adsorption sites in the acidic environment. However, after eight adsorption–desorption regeneration cycles, the Pb^2+^ removal rate remained at approximately 83%, indicating that the IRET-ZEO exhibits excellent acid resistance and regeneration performance. The competitive adsorption and regeneration adsorption results confirm that the IRET-ZEO has substantial potential for practical applications [44].

## 3. Materials and Methods

### 3.1. Materials

NaOH, H_2_SO_4_, Ethylenediaminetetraacetic acid disodium salt (Na_2_EDTA), Pb(NO_3_)_2_, Zn(NO_3_)_2_·6H_2_O, Cr(NO_3_)_3_·9H_2_O, Cd(NO_3_)_2_·4H_2_O, and CuSO_4_ were all purchased from Sinopharm Group and are analytical grade. The IRETs used in the experiments were sourced from a rare earth mine in Changting, Fujian, China, following in situ leaching.

### 3.2. Activation of Tailings and Zeolite Crystallization

In this study, clay minerals from IRETs served as the silicon and aluminum sources for zeolite synthesis. First, the tailings were screened using a 200-mesh Tyler standard sieve (0.074 mm), and the undersized portion was subjected to thermal activation to stimulate the reactivity of the silicon and aluminum components. Specifically, a specific quantity of rare earth tailings was placed in a muffle furnace and heated at a rate of 10 °C/min. The samples were held at different calcination temperatures (550 °C, 650 °C, 750 °C, and 850 °C) for 1 h. After thermal activation, the samples were cooled to room temperature at a rate of 5 °C/min, yielding thermally activated IRETs. A specific quantity of the activated tailings was then placed into a conical flask, and a NaOH solution with varying concentrations (1 mol/L, 3 mol/L, and 5 mol/L) was added, ensuring a liquid-to-solid ratio of 10:1. The beaker was placed on a thermostatic heating magnetic stirrer, and the crystallization process was conducted at different temperatures (45 °C, 65 °C, and 85 °C) for predetermined times (3 h, 8 h, and 10 h). After crystallization, a vacuum pump was used to separate the solid and liquid phases by filtration. The crystallized product was rinsed repeatedly with deionized water until the pH was neutral. Finally, the product was dried in an oven.

### 3.3. Adsorption Experiments

A Pb^2+^ solution with an initial concentration ranging from 100 to 1000 mg/L was prepared for adsorption studies. A 50 mL Pb^2+^ solution was placed in a triangle flask, and 0.01 mol/L NaOH and H_2_SO_4_ solutions were used to adjust the adsorption pH to specific values. A specified mass (0.01 to 0.1 g) of IRET-ZEO was added. The flask was then placed in a thermostatic water bath shaker (150 rpm) where adsorption occurred for a designated time (10 to 90 min) at a controlled temperature of 25 °C. Upon completion of the adsorption process, the supernatant solution was collected and the Pb^2+^ concentration was measured. Three parallel samples were averaged for each group of experiments. The removal efficiency (%) and adsorption capacity (mg/g) of the Pb^2+^ were calculated using Equations (1) and (2), respectively.

(1) $$\eta =\frac{C_{0}-C_{t}}{C_{0}}\times 100\%$$

(2) $$q=\frac{(C_{0}-C_{e})\times V}{m}$$

η: Removal efficiency (%);
C_0_: Initial concentration of Pb^2+^ (mg/L);
C_t_: Concentration of Pb^2+^ present in solution after adsorption time t (mg/L);
C_e_: Concentration of Pb^2+^ remaining in solution at adsorption equilibrium (mg/L);
q: Adsorption capacity (mg/g);
V: Volume of solution used in the adsorption experiment (L);
m: Mass of adsorbent material used (g).

### 3.4. Adsorption Kinetics

The adsorption kinetics of the Pb^2+^ from the solution mixed with the IRET-ZEO were fitted to the pseudo-first-order kinetic equation (Equation (3)), pseudo-second-order kinetic equation (Equation (4)), Elovich kinetic equation (Equation (5)), and the intraparticle diffusion model (Equation (6)). The adsorption kinetic model of Pb^2+^ by the IRET-ZEO was evaluated through the linear correlation coefficient (R^2^) for each model. The expressions of the kinetic models are as follows [45,46,47,48]:

(3) $$\ln(q_{e}-q_{t})=\ln q_{e}-k_{1}t$$

(4) $$\frac{t}{q_{t}}=\frac{1}{k_{2}q_{e}^{2}}+\frac{1}{q_{e}}t$$

(5) $$q_{t}=\beta \ln(\alpha \times \beta )+\beta \ln t$$

(6) $$q_{t}=k_{i}t^{0.5}+C$$

t: Adsorption time (min);
k_1_: Adsorption rate constant obtained from the pseudo-first-order kinetic model (min^−1^);
k_2_: Adsorption rate constant obtained from the pseudo-second-order kinetic model (g·mg^−1^·min^−1^);
q_e_: Adsorption capacity at equilibrium (mg·g^−1^);
q_t_: Adsorption capacity at time t (mg·g^−1^);
α: Elovich coefficient (mg·g^−1^·min^−1^);
β: Elovich coefficient (g·mg^−1^);
k_i_: Adsorption rate constant obtained from the intraparticle diffusion model (mg·g^−1^·min^−1/2^);
C: Constant (mg·g^−1^).

### 3.5. Selective Adsorption Experiments

A mixed solution of metal ions was prepared by dissolving Pb(NO_3_)_2_, Zn(NO_3_)_2_·6H_2_O, Cr(NO_3_)_3_·9H_2_O, Cd(NO_3_)_2_·4H_2_O, and CuSO_4_ in deionized water with the initial concentration of each metal ion set to 100 mg/L. An amount of 0.1 g IRET-ZEO was added to 50 mL of the mixed ion solution and placed in a thermostatic water bath shaker for 10 min at 25 °C. After adsorption, the supernatant was collected and the concentrations of the metal ions were measured. The removal efficiency of each metal ion in the mixed solution was calculated using Equation (1).

### 3.6. Regeneration Adsorption Experiment

The regeneration experiments were carried out under optimal adsorption conditions (pH was 4.0, adsorbent dosage was 0.4 g/L, contact time was 5 min, and initial concentration of Pb^2+^ was 100 mg/L). The desorption agent of the lead ions adsorbed on the zeolite was a 0.5 mol/L Na_2_EDTA solution, the desorption time was 60 min, and the liquid–solid ratio was kept consistent with the adsorption experiments. The desorbed zeolite was washed to neutral with deionized water and then dried to constant weight at 100 °C in an oven. The regenerated zeolite was used for the next adsorption–desorption cycle experiment.

### 3.7. Characterization Methods

#### 3.7.1. X-Ray Diffraction Analysis (XRD)

An X-Ray diffraction (XRD) analysis was conducted using an Ultima IV X-Ray diffractometer for phase identification of the samples at scanning ranges from 5° to 80°. The Cu target was used as the target material, and the voltage and current were set to 25 mA and 40 kV, respectively.

#### 3.7.2. Scanning Electron Microscope (SEM)

The microstructure of the samples was examined using an SU8020 scanning electron microscope (SEM) (Thermo Fisher Scientific Inc., Waltham, MA, USA). The IRET-ZEO was first dried in a vacuum drying oven, and the sample was then mounted on conductive tape and coated with gold prior to SEM observation. The morphology and microstructure of the IRET-ZEO were observed using SEM.

#### 3.7.3. Inductively Coupled Plasma Emission Spectrometer (ICP-OES)

The concentration of Pb^2+^ and other metal ions in the solution before and after adsorption was measured using an Optima 8000 ICP-OES spectrometer (PerkinElmer, Inc., Waltham, MA, USA). The operating conditions were set as follows: cooling gas flow rate at 17.0 L/min, solution uptake rate at 2 mL/min, and observation height at 17 mm.

#### 3.7.4. X-Ray Fluorescence Spectrometer (XRF)

The main chemical composition of the IRETs was analyzed by PANalytical Axios. Ag was used as the target material, and a test voltage of 50 kV and a current of 100 μA were selected.

## 4. Conclusions

In this study, IRETs were used as raw materials, and a classification activation method combined with thermal activation was applied to activate the tailings. A mild alkali dissolution process was then employed to synthesize zeolite materials. The effects of thermal activation temperature, crystallization temperature, alkali concentration, and crystallization time on the phase composition of the zeolite were investigated. The synthesized IRET-ZEO was then tested for its adsorption performance of Pb^2+^ in aqueous solutions. The main conclusions are as follows:

(1) The primary mineral phases of the IRETs are kaolinite, quartz, and illite. The tailings contain 61.60% SiO_2_ and 24.86% Al_2_O_3_, with lower concentrations of other impurities. IRETs are a potential source of silica–aluminum for zeolite preparation. The clay minerals used in this study were primarily concentrated in particles smaller than 0.074 mm, and the clay-rich components could be easily separated by sieving. However, further work is required to explore the resource utilization of the coarse, silica-rich components.
(2) Based on a phase analysis of the crystallization products, the optimal conditions for synthesizing the IRET-ZEO from the tailings through alkali dissolution were identified. The optimal activation parameter was calcination at 850 °C for 1 h. The optimal crystallization parameters were a NaOH concentration of 5 mol/L, a crystallization time of 3 h, and a crystallization temperature of 85 °C. The synthesized product was cubic A-type zeolite.
(3) In acidic solutions with a pH range of 2 to 5.5 and an initial Pb^2+^ concentration of 100 mg/L, the removal rate of Pb^2+^ by the IRET-ZEO remained consistently above 96.7%. Notably, when the solution pH exceeded 4.2, the Pb^2+^ removal rate reached 100%. This demonstrated that the IRET-ZEO exhibited a fast adsorption rate (reaching equilibrium within 5 min), a high adsorption capacity (378.35 mg/g), and excellent acid resistance, selectivity, and regeneration performance. The adsorption kinetics results indicated that the adsorption of Pb^2+^ by the IRET-ZEO follows a pseudo-second-order kinetic model, with the process being primarily governed by chemical interactions. This work provides a new strategy for the resource and valorization utilization of IRETs with “treating waste with waste” applications.

## Figures and Tables

**Table 1:** Main chemical composition of IRETs.

SiO_2_	Al_2_O_3_	MgO	CaO	K_2_O	Na_2_O	Fe_2_O_3_	SO_3_	Rb_2_O	Y_2_O_3_	LOI
61.60	24.86	0.32	0.09	4.41	0.39	1.95	0.21	0.10	0.05	6.02

LOI: lost on ignition.

**Table 2:** Particle size distribution of IRETs.

Particle Size/mm	Mass/g	Distribution Ratio/%
>0.8	114.00	57.00
0.074~0.8	48.12	24.09
0.044~0.074	4.88	2.44
<0.044	32.94	16.47
Total	200.00	100.00

**Table 3:** Comparison between the results of this work and others found in the literature.

Adsorbents	pH	Dosage (g/L)	Adsorption Equilibrium Time (min)	Maximum Adsorption Capacity (mg/g)	References
Chitosan–zeolite	4.5	5	1440	275	[35]
LFS + FA	5	1	180	292.8	[36]
MZA	4~5	-	120	330.72	[37]
Zeolite NaY	6	1	2880	527.12	[38]
Metakaolin-based geopolymer	4.0	-		147.06	[39]
HCl-treated Egyptian kaolin	5.5	-	90	34.5	[40]
Na-T13	8	0.2	5	170.12	[41]
MoS_2_@kaolin	2~5.5	1.6	40	280.39	[42]
IRET-ZEO	2~5.5	0.4	5	378.35	This work

**Table 4:** Kinetic parameters of Pb^2+^ adsorption by IRET-ZEO.

Models	Kinetic Parameters		
Pseudo-first-order kinetic model	k_1_ (min^−1^) 0.0267	q_e_ (mg·g^−1^) 0.18	R^2^ 0.2393
Pseudo-second-order kinetic model	k_2_ (g·mg^−1^·min^−1^) 1.1532	q_e_ (mg·g^−1^) 53.76	R^2^ 0.9999
Intraparticle diffusion model	k_i_ (mg·g^−1^·min^−1/2^) 0.6634	C (mg·g^−1^) 52.28	R^2^ 1.0000
	0.0116	53.79	0.5734
Elovich equation	α (mg·g^−1^·min^−1^) -	β (mg·g^−1^) 0.18	R^2^ 0.6067

## Data Availability

The data that support the findings of this study are available from the corresponding author upon reasonable request.

## References

[1] Italiya G., Ahmed M.H., Subramanian S. (2022). Titanium oxide bonded Zeolite and Bentonite composites for adsorptive removal of phosphate. Environ. Nanotechnol. Monit. Manag..

[2] Vardhan K.H., Kumar P.S., Panda R.C. (2019). A review on heavy metal pollution, toxicity and remedial measures: Current trends and future perspectives. J. Mol. Liq..

[3] Yang X., Liu L.H., Wang Y., Lu T., Wang Z.W., Qiu G.H. (2023). Sustainable and reagent-free cathodic precipitation for high-efficiency removal of heavy metals from soil leachate. Environ. Pollut..

[4] Lin H., Zhou M.Y., Li B., Dong Y.B. (2023). Mechanisms, application advances and future perspectives of microbial-induced heavy metal precipitation: A review. Int. Biodeter. Biodegr..

[5] Fei Y.H., Hu H.Y. (2023). Recent progress in removal of heavy metals from wastewater: A comprehensive review. Chemosphere.

[6] Liu Y., Fu R.Q., Sun Y., Zhou X.X., Baig S.A., Xu X.H. (2016). Multifunctional nanocomposites Fe_3_O_4_@SiO_2_-EDTA for Pb(II) and Cu(II) removal from aqueous solutions. Appl. Surf. Sci..

[7] Parry D., Jabez W.O., Kirubhadharsini B.L. (2024). An insight on the plausible biological and non-biological detoxification of heavy metals in tannery waste: A comprehensive review. Environ. Res..

[8] Cao Y.Y., Xiao W.H., Shen G.H., Ji G.Y., Zhang Y., Gao C.F., Han L.J. (2019). Carbonization and ball milling on the enhancement of Pb(II) adsorption by wheat straw: Competitive effects of ion exchange and precipitation. Bioresour. Technol..

[9] Oberta A., Wasilewski J., Wódzki R. (2011). Structure and transport properties of polymer inclusion membranes for Pb(II) separation. Desalination.

[10] Liang B., Zhu P., Gu J., Yuan W., Xiao B., Hu H., Rao M. (2024). Advancing Adsorption and Separation with Modified SBA-15: A Comprehensive Review and Future Perspectives. Molecules.

[11] Peng Y., Zhu P., Zou Y., Gao Q., Xiong S., Liang B., Xiao B. (2024). Overview of Functionalized Porous Materials for Rare-Earth Element Separation and Recovery. Molecules.

[12] Liang B., Hu H., Xiao B., Lu Z., Yuan W., Huang Z. (2024). Water Leaching Kinetics of Boron from the Alkali-Activated Ludwigite Ore. Molecules.

[13] Liang B., Gu J., Zeng X., Yuan W., Rao M., Xiao B., Hu H. (2024). A Review of the Occurrence and Recovery of Rare Earth Elements from Electronic Waste. Molecules.

[14] Maghfirah A., Ilmi M.M., Fajar A.T.N., Kadja G.T.M. (2020). A review on the green synthesis of hierarchically porous zeolite. Mat. Today Chem..

[15] Yoldi M., Fuentes-Ordoñez E.G., Korili S.A., Gil A. (2019). Zeolite synthesis from industrial wastes. Micropor. Mesopor. Mat..

[16] Abbadi A., Mucsi G. (2024). A review on complex utilization of mine tailings: Recovery of rare earth elements and residue valorization. J. Environ. Chem. Eng..

[17] Han C.Y., Yang J., Dong S.L., Ma L.Q., Dai Q.X., Guo J.Y. (2025). Zeolite preparation from industrial solid waste: Current status, applications, and prospects. Sep. Purif. Technol..

[18] Flores C.G., Schneider H., Dornelles J.S., Gomes L.B., Marcilio N.R., Melo P.J. (2021). Synthesis of potassium zeolite from rice husk ash as a silicon source. Clean. Eng. Technol..

[19] Cao C.Y., Xuan W.W., Yan S.Y., Wang Q. (2023). Zeolites synthesized from industrial and agricultural solid waste and their applications: A review. J. Environ. Chem. Eng..

[20] Li W.L., Jin H.X., Xie H.Y., Ma L.R. (2023). Utilization of electrolytic manganese residue and bauxite to synthesize zeolite a for pickle liquor adsorption: Characterization, mechanisms and performance. J. Clean. Prod..

[21] Valbuena H.M.G., Medinaa A.F., Vargasa J.C., Fandiño O.H. (2023). Synthesis of zeolites Na-A, Na-X, and analcime from crushed stone waste and their applications in heavy metal removal in aqueous media. Chem. Eng. Res. Des..

[22] Sayehi M., Delahay G., Tounsi H. (2022). Synthesis and characterization of ecofriendly materials zeolite from waste glass and aluminum scraps using the hydrothermal technique. J. Environ. Chem. Eng..

[23] Lin Y.J., Chen J. (2021). Resourcization and valorization of waste incineration fly ash for the synthesis of zeolite and applications. J. Environ. Chem. Eng..

[24] Lee W.H., Lin Y.W., Lin K.L. (2023). Optimization of synthesis parameters for the preparation of zeolite by reusing industrialwaste as raw material: Sandblasting waste and solar panel waste glass. Solid State Sci..

[25] Lv Y.W., Ma B.Z., Liu Y.B., Wang C.Y., Chen Y.Q. (2022). Adsorption behavior and mechanism of mixed heavy metal ions by zeolite adsorbent prepared from lithium leach residue. Micropor. Mesopor. Mat..

[26] Indira V., Abhitha K. (2022). A review on recent developments in Zeolite A synthesis for improved carbon dioxide capture: Implications for the water-energy nexus. Energy Nexus.

[27] Gao S., Peng H., Song B., Zhang J.X., Wu W.X., Vaughan J., Zardo P., Vogrin J., Tulloch S., Zhu Z.H. (2023). Synthesis of zeolites from low-cost feeds and its sustainable environmental applications. J. Environ. Chem. Eng..

[28] Qiang Z.Q., Shen X.J., Guo M., Cheng F.Q., Zhang M. (2019). A simple hydrothermal synthesis of zeolite X from bauxite tailings for highly efficient adsorbing CO2 at room temperature. Micropor. Mesopor. Mat..

[29] Li J.F., Xiao Y.F., Feng X.J., Wang J., Ma Z.Y., Yao R.F., Zhai Y.Q., Tian L. (2024). Leaching of ion adsorption rare earths and the role of bioleaching in the process: A review. J. Clean. Prod..

[30] Zhou F., Zhang Y.X., Liu Q., Huang S.H., Wu X.Y., Wang Z.W., Zhang L.S., Chi R. (2023). Modified tailings of weathered crust elution-deposited rare earth ores as adsorbents for recovery of rare earth ions from solutions: Kinetics and thermodynamics studies. Miner. Eng..

[31] He Q., Chen J.F., Gan L.M., Gao M.L., Zan M.M., Xiao Y.F. (2023). Insight into leaching of rare earth and aluminum from ion adsorption type rare earth ore: Adsorption and desorption. J. Rare Earth..

[32] Yang Y.J., Zhang M.Q., Feng L., Huang B., Zhai R.Y., Sun X.Q. (2024). Sustainable reutilization of ion-adsorbed rare earth tailings: Preparation of low-cost functionalized pigments. Ceram. Int..

[33] Cheng J.C., Hua X.L., Zhang G.H., Yu M.Q., Wang Z., Zhang Y.L., Liu W., Chen Y.J., Wang H.M., Luo Y.D. (2024). Synthesis of high-crystallinity Zeolite A from rare earth tailings: Investigating adsorption performance on typical pollutants in rare earth mines. J. Hazard. Mater..

[34] Ismail U.M., Vohra M.S., Onaizi S.A. (2024). Adsorptive removal of heavy metals from aqueous solutions: Progress of adsorbents development and their effectiveness. Environ. Res..

[35] Şenol Z.N.M., Elma E., Messaoudi N.E., Mehmeti V. (2023). Performance of cross-linked chitosan-zeolite composite adsorbent for removal of Pb^2+^ ions from aqueous solutions: Experimental and Monte Carlo simulations studies. J. Mol. Liq..

[36] Ma W.Q., Yi Y.R., Fang M.H., Lin Y., Li C.H., Li J., Liu W. (2024). Zeolite prepared from high-calcium ladle furnace slag and fly ash for Pb^2+^ removal. J. Water Process Eng..

[37] Cui K.B., Lyu J.W., Liu H.Z., Yang J.L., Yan Z.Q., Yang W., Liu X., Qiu J. (2024). Eco-friendly synthesis of magnetic zeolite A from red mud and coal gasifcation slag for the removal of Pb^2+^ and Cu^2+^. J. Environ. Chem. Eng..

[38] Shichalin O.O., Papynov E.K., Ivanov N.P., Balanov M.I., Dran’kov A.N., Shkuratov A.L., Zarubina N.V., Fedorets A.N., Mayorov V.Y., Lembikov A.O. (2024). Study of adsorption and immobilization of Cs^+^, Sr^2+^, Co^2+^, Pb^2+^, La^3+^ ions on Na-Faujasite zeolite transformed in solid state matrices. Sep. Purif. Technol..

[39] Cheng T.W., Lee M.L., Ko M.S., Ueng T.H., Yang S.F. (2012). The heavy metal adsorption characteristics on metakaolin-based geopolymer. Appl. Clay Sci..

[40] Drweesh S.A., Fathy N.A., Wahba M.A., Hanna A.A., Akarish A.I.M., Elzahany E.A.M., El-Sherif I.Y., Abou-El-Sherbini K.S. (2016). Equilibrium, kinetic and thermodynamic studies of Pb(II) adsorption from aqueous solutions on HCl-treated Egyptian kaolin. J. Environ. Chem. Eng..

[41] Gao S., Liu Y.H. (2022). Potassium-assisted synthesis of SUZ-4 zeolite as an efficient adsorbent for Pb^2+^ removal from wastewater. Sep. Purif. Technol..

[42] Yuan W.Q., Kuang J.Z., Yu M.M., Huang Z.Y., Zou Z.L., Zhu L.P. (2021). Facile preparation of MoS_2_@Kaolin composite by one-step hydrothermal method for efficient removal of Pb(II). J. Hazard. Mater..

[43] Yuan W.Q., Kuang J.Z., Yu M.M., Huang Z.Y., Wang X.Y., Xiao J.J., Zhang S.Y., Cheng H., Yang Y.Q. (2022). Facile synthesis and characterization of ZnS polymorphs/Halloysite composite for efficiently selective adsorption of Al(III) from acidic rare earth ions solution. Sep. Purif. Technol..

[44] Yuan W.Q., Kuang J.Z., Hu H.X., Ding D., Yu M.M. (2024). Preparation of chitosan mesoporous membrane/halloysite composite for efficiently selective adsorption of Al(III) from rare earth ions solution through constructing pore structure on substrate. Int. J. Biol. Macromol..

[45] Ho Y.S., McKay G. (1999). Comparative sorption kinetic studies of dye and aromatic compounds onto fly ash. J. Environ. Sci. Health Part A.

[46] Ho Y.S., McKay G. (1999). Pseudo-second order model for sorption processes. Process Biochem..

[47] Bhattacharyya K.G., Gupta S.S. (2006). Pb(II) uptake by kaolinite and montmorillonite in aqueous medium: Influence of acid activation of the clays. Colloid. Surface A.

[48] Gong F.Z., Cai H.M., Zhou B., Ou H.L. (2018). The synthesis and characterization of AlPO4 hollow microspheres of uniform size, and the sorption properties for Pb^2+^, Cd^2+^, Cu^2+^, and Zn^2+^. Colloid. Surface A.

